# Supplementary material for: Membrane-localized expression, production and assembly of Vibrio parahaemolyticus T3SS2 provides evidence for transertion
Source: Nat Commun. 2023 Mar 2;14:1178. doi: 10.1038/s41467-023-36762-z (PMC9977878; doi:10.1038/s41467-023-36762-z)
Supplement: Supplementary file 1 — Supplementary Information [file 41467_2023_36762_MOESM1_ESM.pdf]

## Supplementary Information for

# Membrane-localized expression, production and assembly of *Vibrio parahaemolyticus* T3SS2 provides evidence for transertion

Karan Gautam Kaval[1,2], Suneeta Chimalapati[4], Sara Siegel[5], Nalleli Garcia Rodriguez[6], Jananee Jaishankar[1], Ankur B. Dalia[7] & Kim Orth[1,2,3*]

**Affiliations:**

[1]Department of Molecular Biology, University of Texas Southwestern Medical Center, Dallas, TX 75390, USA,

[2]Howard Hughes Medical Institute, University of Texas Southwestern Medical Center, Dallas, TX 75390, USA

[3]Department of Biochemistry, University of Texas Southwestern Medical Center, Dallas, TX 75390, USA

[4]Reata Pharmaceuticals, Plano, TX 75204, USA

[5]Biomanufacturing Training and Education Center, North Carolina State University, Raleigh, NC 27606

[6]Department of Microbiology and Cell Science, University of Florida, Gainesville, FL 32611, USA

[7]Department of Biology, Indiana University, Bloomington, IN 47405, USA

***corresponding author**: Kim Orth

**Email:** kim.orth@utsouthwestern.edu

**This PDF file includes:**

Supplementary Table 1

Supplementary Figs. 1-7

**Supplementary Table 1. Bacterial strains, plasmids and primers.**

| | Relevant characteristics | Source/ Reference |
|---|---|---|
| **Strains** | | |
| ***V. parahaemolyticus*** | | |
| CAB2 | Reference strain, RIMD2210633 Δ*tdh* Δ*trh* Δ*exsA* | [1] |
| CAB3 | RIMD2210633 Δ*tdh* Δ*trh* Δ*vtrA/vtrC* | [1] |
| CAB4 | RIMD2210633 Δ*tdh* Δ*trh* Δ*exsA* Δ*vtrA/vtrC* | [1] |
| SS2B1 | CAB2, $P_{araBAD}$-*ygfp-parBMT1*, $Km^R$ | This work |
| SS2B3 | CAB2 1.42 Mbp::*parSMT1* | This work |
| SS2B5 | CAB2 1.42 Mbp::*parSMT1*, $P_{araBAD}$-*ygfp-parBMT1*, $Km^R$ | This work |
| SS1H4 | CAB2 Δ*vpa1343::vpa1343-kpnI* | This work |
| SS2B8 | CAB2 Δ*vpa1343::vpa1343S3C-kpnI* | This work |
| SS1G6 | CAB2 Δ*vpa1343::vpa1343S32C-kpnI* | This work |
| SS1I6 | CAB2 Δ*vpa1343::vpa1343S62C-kpnI* | This work |
| SS1H5 | CAB2 Δ*vpa1343::vpa1343S85C-kpnI* | This work |
| SS1I7 | CAB2 Δ*vpa1343::vpa1343S91C-kpnI* | This work |
| SS2D4 | CAB2 Δ*vpa1343::vpa1343S3C-kpnI,* 1.42 Mbp::*parSMT1* | This work |
| VPKK6 | CAB2 Δ*vpa1343::vpa1343S3C-kpnI,* 1.42 Mbp::*parSMT1*, $P_{araBAD}$-*ygfp-parBMT1*, $Km^R$ | This work |
| SC279 | CAB2 Δ*vpa1343::vpa1343S3C-kpnI,* 1.42 Mbp::*parSMT1,* 0.458 Mbp::*parSP1* | This work |
| VPKK11 | CAB2 Δ*vpa1343::vpa1343S3C-kpnI,* 1.42 Mbp::*parSMT1,* 0.458 Mbp::*parSP1*, $P_{araBAD}$-*cfp-parBP1-ygfp-parBMT1*, $Km^R$ | This work |
| SC288 | CAB2 Δ*vpa1343::vpa1343S3C-kpnI,* 1.42 Mbp::*parSMT1,* Δ*vpa1348::vpa1348-msfgfp* | This work |
| SC295 | CAB2 Δ*vpa1343::vpa1343S3C-kpnI,* 1.42 Mbp::*parSMT1,* Δ*vpa1348::vpa1348-msfgfp*, $P_{araBAD}$-*cfp-parBMT1*, $Km^R$ | This work |
| JJ1 | CAB2 Δ*vpa1343::vpa1343S3C-kpnI,* 1.42 Mbp::*parSMT1,* 0.458 Mbp::*parSP1*-$P_{vpa1348}$ | This work |
| VPKK15 | CAB2 Δ*vpa1343::vpa1343S3C-kpnI,* 1.42 Mbp::*parSMT1,* 0.458 Mbp::*parSP1*-$P_{vpa1348}$, $P_{araBAD}$-*cfp-parBP1-ygfp-parBMT1*, $Km^R$ | This work |
| VPKK16 | CAB2 Δ*vpa1343::vpa1343S3C-kpnI,* 1.42 Mbp::*parSMT1,* Δ*vpa1348::vpa1348-msfgfp,* Δ*vpa1357::vpa1357-ms2SL* | This work |
| VPKK17 | CAB3 1.42 Mbp::*parSMT1* | This work |
| VPKK18 | CAB3 1.42 Mbp::*parSMT1*, $P_{araBAD}$-*ygfp-parBMT1*, $Km^R$ | This work |
| VPKK19 | CAB2 Δ*vpa1343::vpa1343S3C-kpnI,* 1.42 Mbp::*parSMT1,* Δ*vpa1348::vpa1348-msfgfp,* Δ*vpa1357::vpa1357-ms2SL*, $P_{araBAD}$-*mcp-ecfp*, $Km^R$ | This work |
| | | |
| ***V. cholerae*** | | |
| TND1379 | 0.11 Mbp::*parSP1,* 1.963 Mbp::*parSMT1,* Δ*lacZ*::$P_{lac}$-*CFP-parBP1 yGFP-parBMT1,* $Zeo^R$ | [2] |

| | | |
|---|---|---|
| ***E. coli*** | | |
| DH5α (pRK2073) | Strain carrying the conjugation helper plasmid, pRK2073 | Lab stock |
| Mach1 | Host for cloning vectors | Lab stock |
| S17-1 | Host for pDM4-based plasmids, *λ pir*$^+$ | Lab stock |
| Plasmids | | |
| pUC18 | Cloning vector, Amp$^R$ | Lab stock |
| pDM4 | Suicide vector, *γ ori R6K*, *sacB*, Cm$^R$ | Lab stock |
| pC014-*lwcas13a-msfgfp* | msfGFP, Amp$^R$ | [3] |
| pDZ415 | 24MS2SL, Amp$^R$ | [4] |
| pDZ274 | MCP, Amp$^R$ | [4] |
| pLAU53 | $P_{araBAD}$, Amp$^R$ | [5] |
| pLAU(Kan) | Amp$^R$ cassette replaced with Km$^R$ cassette of pET28a, Km$^R$ | This work |
| SS2A5 | pLAU(Kan) [$P_{araBAD}$-*ygfp-parBMT1*], Km$^R$ | This work |
| SS1I3 | CAB2 1.42 Mbp-*parSMT1* cloned into pTWIST by gene synthesis | This work |
| SS1I9 | pDM4 [1.42 Mbp-*parSMT1*], Cm$^R$ | This work |
| ECKK7 | pLAU(Kan) [$P_{araBAD}$-*cfp-parBP1-ygfp-parBMT1*], Km$^R$ | This work |
| SC299 | pDM4 [+/- 1 Kbp *vpa1348-msfgfp*], Cm$^R$ | This work |
| SC300 | pDM4 [0.458 Mbp-*parSP1*], Cm$^R$ | This work |
| SC309 | pLAU(Kan) [$P_{araBAD}$-*cfp-parBMT1*], Km$^R$ | This work |
| SS1F6 | pUC18 [+/- 3 Kbp *vpa1343*], Amp$^R$ | This work |
| SS1F7 | pUC18 [+/- 3 Kbp *vpa1343-kpnI*], Amp$^R$ | This work |
| SS1G7 | pDM4 [+/- 1.3 Kbp *vpa1343-kpnI*], Cm$^R$ | This work |
| SS2A1 | pDM4 [+/- 1.3 Kbp *vpa1343S3C-kpnI*], Cm$^R$ | This work |
| SS1G1 | pDM4 [+/- 1.3 Kbp *vpa1343S32C-kpnI*], Cm$^R$ | This work |
| SS2H7 | pDM4 [+/- 1.3 Kbp *vpa1343S62C-kpnI*], Cm$^R$ | This work |
| SS1G8 | pDM4 [+/- 1.3 Kbp *vpa1343S85C-kpnI*], Cm$^R$ | This work |
| SS2H8 | pDM4 [+/- 1.3 Kbp *vpa1343S91C-kpnI*], Cm$^R$ | This work |
| ECKK11 | pDM4 [+/- 800 bp *vpa1357-24ms2SL*], Cm$^R$ | This work |
| ECKK12 | pLAU(Kan) [$P_{araBAD}$-*mcp-ecfp*], Km$^R$ | This work |
| NG56 | pDM4 [0.458 Mbp-*parSP1*-$P_{vpa1348}$], Cm$^R$ | This work |
| | | |
| **Primers** | | |
| F/pET28a_KmR_KpnI | 5'-AAAAGGTACCATCCTTTGATCTTTTCTACGGGGTCT-3' | This work |

| | | |
|---|---|---|
| R/pET28a_KmR_SpeI | 5’-AAAAACTAGTGAATTAATTCTTAGAAAAACTCATCGAGCATC-3’ | This work |
| Lf/pLAU53_nobla_KpnI | 5’-AAAAGGTACCAGAGTTTGTAGAAACGCAAAAAGGC-3’ | This work |
| Ri/pLAU53_nobla_SpeI | 5’-AAAAACTAGTCTGTCAGACCAAGTTTACTCATATATACTTTAG-3’ | This work |
| F/ygfp-parBMT1_NheI | 5’- AAAAGCTAGCAGGAGGAATTCACC-3’ | This work |
| R/parBMT1_HindIII | 5’-AAAAAAGCTTTTACTCACCTGATTCTGGAAGTC-3’ | This work |
| KK13 | 5’-TTCCAGAATCAGGTGAGTAAAAGCTTGGCTGTTTTGGCGGA-3’ | This work |
| KK14 | 5’-AGTTCTTCTCCTTTACTCATGTGAATTCCTCCTGCTAGAGAGCT-3’ | This work |
| KK15 | 5’-CTCTAGCAGGAGGAATTCACATGAGTAAAGGAGAAGAACTTTTCACTGG-3’ | This work |
| KK16 | 5’-CCGCCAAAACAGCCAAGCTTTTACTCACCTGATTCTGGAAGTCTTTCC-3’ | This work |
| KK21 | 5’-CCGGGTACCATTTTATTTCTGGCGTGGCTAGTTGTTGATTGG-3’ | This work |
| KK22 | 5’-CGAAAATCAAGCTTAGCATGCATGGCTATAATAGTACTTGAGAAGGAGGCA-3’ | This work |
| KK23 | 5’-CTAGTCTAGATTATGACGTGTATTTCATTATCGATTTTAAATCAAGAACAAG-3’ | This work |
| KK28 | 5’-CATGCCATGGCTATAATAGTACTTGAGAAGGAGGCA-3’ | This work |
| KK37 | 5’-CGACAATCATGGCCCCACTAGTGACGCGTACT-3’ | This work |
| KK38 | 5’-GTGTGTACGCCTTCTAGATAGATCTTGCATGCGGGTAACCTGAG-3’ | This work |
| KK40 | 5’-CTAGAGCGGCCTATGATACAAGCTTAAGATTACTAATTATTGCTGGTTTAATCGG-3’ | This work |
| KK41 | 5’-TGTATCATAGGCCGCTCTAGAACTAGTGGATCCTACGG-3’ | This work |
| KK42 | 5’-CCTTATATCAGCAGCCCGGGGGATCTGATGA-3’ | This work |
| KK43 | 5’-CCCGGGCTGCTGATATAAGGTCAAATTAAATGAAAAAAGACTAAATCAGTATCGTTCAGG-3’ | This work |
| KK45 | 5’-GGTTCAACAAAATAATCGACGCGTCTGCAGAAG-3’ | This work |
| KK46 | 5’-AAAGAAGGATGTTTTCGTCATATGGATCCGATATCGCCG-3’ | This work |
| KK49 | 5’-CTGCAGACGCGTCGATTATTTTGTTGAACCTTTTTGATCAATTCCATAAGGTAAAAC-3’ | This work |
| KK52 | 5’-CGGATCCATATGACGAAAACATCCTTCTTTCTTTTATTCTGAAGTGAATCAC-3’ | This work |
| KK53 | 5’-ATTAGGCGCATTTCTGAACCGACTTCTCCTTTTTCG-3’ | This work |
| KK54 | 5’-GAACCGCAATAAGAAGGATATGGATCTGGAGCTGTAATATAAAAAC-3’ | This work |
| KK55 | 5’-TATCCTTCTTATTGCGGTTCCTACAACTAGTGGAACT-3’ | This work |
| KK56 | 5’-CCCACATTATCTGTCAATCGCTTATAAGATAGAATTTAAAATTTTTGTCACTTCTGC-3’ | This work |
| KK57 | 5’-CGATTGACAGATAATGTGGGTAGCACAACGGCGT-3’ | This work |

| | | |
|---|---|---|
| KK58 | 5’-GGTTCAGAAATGCGCCTAATTTTTTAGCTTTGTCCG-3’ | This work |
| KK59 | 5’-ACTGTGAAAAGGGCTCAGCGCAATAGGAGCTCGTTTTTATATGAATCGTCAAAG-3’ | This work |
| KK60 | 5’-ATAAAAGGGGCATAGCCCCTAGGTGAAATCGTGGCGATTTCAC-3’ | This work |
| KK61 | 5’-AAATCGCCACGATTTCACCTAGGGGCTATGCCCCTTTTATTTTTAAT-3’ | This work |
| KK62 | 5’-ATAAAAACGAGCTCCTATTGCGCTGAGCCCTTTTCACAG-3’ | This work |
| KK66 | 5’-TGGACGAGCTGTACAAGTAAAAGCTTGGCTGT-3’ | This work |
| KK67 | 5’-AACTGAGTAAAGTTAGAAGCATGGAGAAACAGTAGAGAGTTGCGA-3’ | This work |
| KK68 | 5’-ACTCTCTACTGTTTCTCCATGCTTCTAACTTTACTCAGTTCGTTCTCG-3’ | This work |
| KK69 | 5’-AGCTCCTCGCCCTTGCTCACGTCGACGGTATCGATAAGCTTGA-3’ | This work |
| KK70 | 5’-AGCTTATCGATACCGTCGACGTGAGCAAGGGCGAGGAGCT-3’ | This work |
| KK71 | 5’-CCGCCAAAACAGCCAAGCTTTTACTTGTACAGCTCGTCCATGCC-3’ | This work |
| KK72 | 5’-GCGCAGATCTGCGTACACACGTCGCAGGGC-3’ | This work |
| KK73 | 5’-GCGCCTCGAGATGATTGTCGGTAATAAAAACGCAATCATCAC-3’ | This work |
| F/pUC18-1343_BamHI | 5’-AAAAAGGATCCGACTATGTCGATATGAGTAGTTTTGTAAAAGCTGTC-3’ | This work |
| R/pUC18-1343_EcoRI | 5’-AAAAAGAATTCCCTCATTATCATCTTCATCTAGAGACTCCTCAAC-3’ | This work |
| F/sdm1343_KpnI | 5’-AGCTCCAATCGACCATTGGTACCTGCTTTGTCATG-3’ | This work |
| R/sdm1343_KpnI | 5’-CATGACAAAGCAGGTACCAATGGTCGATTGGAGCT-3’ | This work |
| F/sdm1343_S3C | 5'-ACCTGCACCAGCGTTACATAACATATAAAAATCTCCTATAGCA-3' | This work |
| R/sdm1343_S3C | 5'-TGCTATAGGAGATTTTTATATGTTATGTAACGCTGGTGCAGGT-3' | This work |
| F/sdm1343_S32C | 5'-CTTATCAGCTTCTTTAATCAAACATTCGAAAGAAACACCAACCTCTG-3' | This work |
| R/sdm1343_S32C | 5'-CAGAGGTTGGTGTTTCTTTCGAATGTTTGATTAAAGAAGCTGATAAG-3' | This work |
| F/sdm1343_S62C | 5'-CTTTCTGCCGGGGAATGTTTGCAGCTCCAAC-3' | This work |
| R/sdm1343_S62C | 5'-GTTGGAGCTGCAAACATTCCCCGGCAGAAAG-3' | This work |
| F/sdm1343_S85C | 5'-AACTGGTACTGCTACGTTGAAGTGCATTAAAGATTCTATCTCTTCAGC-3' | This work |
| R/sdm1343_S85C | 5'-GCTGAAGAGATAGAATCTTTAATGCACTTCAACGTAGCAGTACCAGTT-3' | This work |
| F/sdm1343_S91C | 5'-GTTGAAGTCAATTAAAGATTCTATCTGTTCAGCAGCACGTAACATC-3' | This work |
| R/sdm1343_S91C | 5'-GATGTTACGTGCTGCTGAACAGATAGAATCTTTAATTGACTTCAAC-3' | This work |

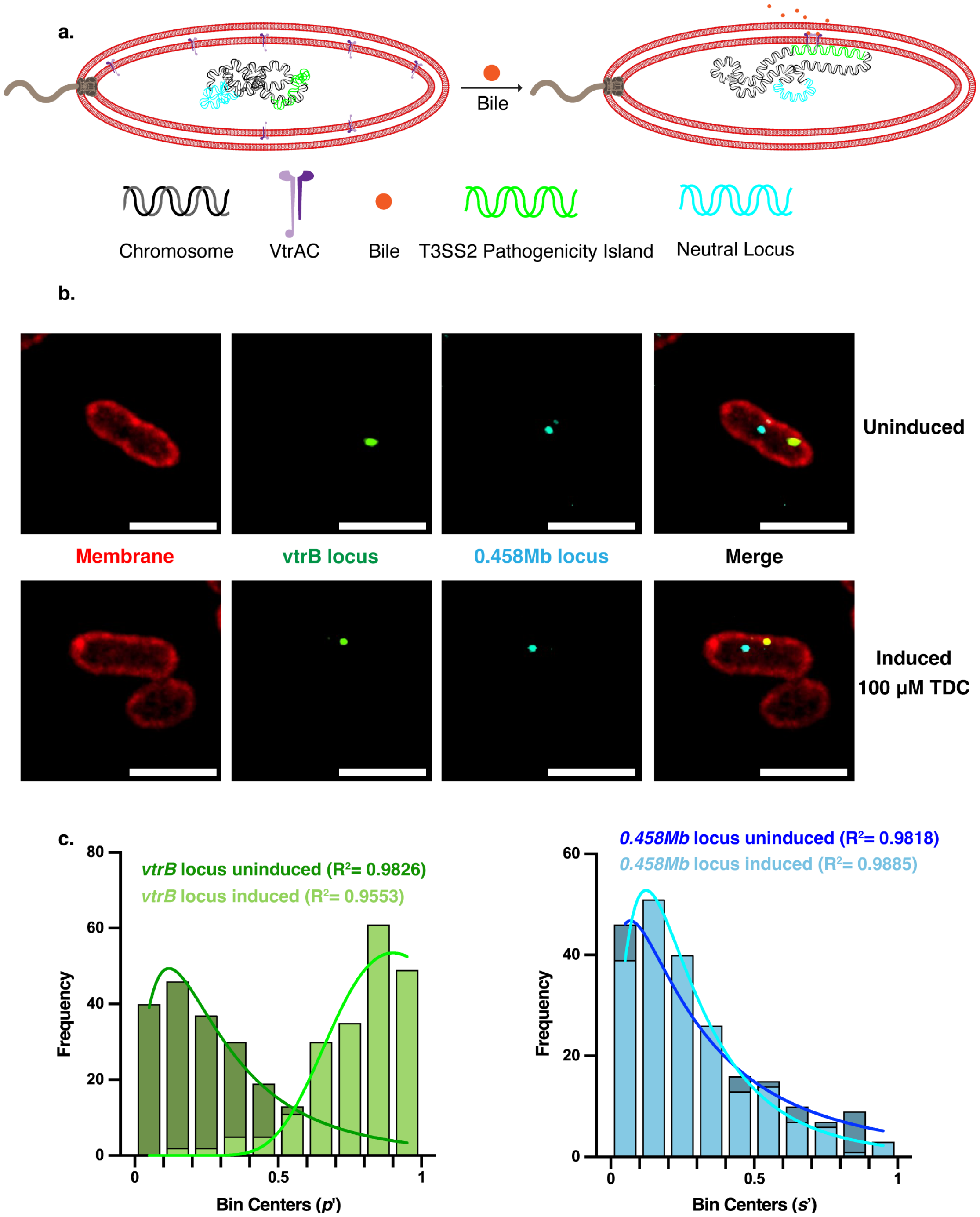


**Supplementary Fig. 1. Bile-induced genomic target capture at the membrane is specific to the *vtrB* locus.**

**a.** Illustration depicting specific capture of the *vtrB* genomic locus, along with the T3SS2 pathogenicity island, at the membrane by bile-activated dimeric VtrA/VtrC, without similarly recruiting a neutral locus lying outside this region. **b.** Confocal micrographs of *V. parahaemolyticus* VPKK11 cells cultured in either non-inducing or inducing (100 $\mu$M TDC) conditions, displaying localization of the *vtrB* (green) and neutral (cyan) loci relative to the membrane (red). Scale bar = 2 $\mu$m. **c.** Frequency distribution plots with nonlinear regression

(lognormal) analyses of normalized *vtrB* loci (p') and neutral loci (s') distances in *V. parahaemolyticus* VPKK11 cells, cultured as described above. N = 200 loci/condition. $R^2$ = goodness of fit of the regression curves. **b** & **c.** Three independent biological repetitions were performed. Source data are provided as a Source Data file.

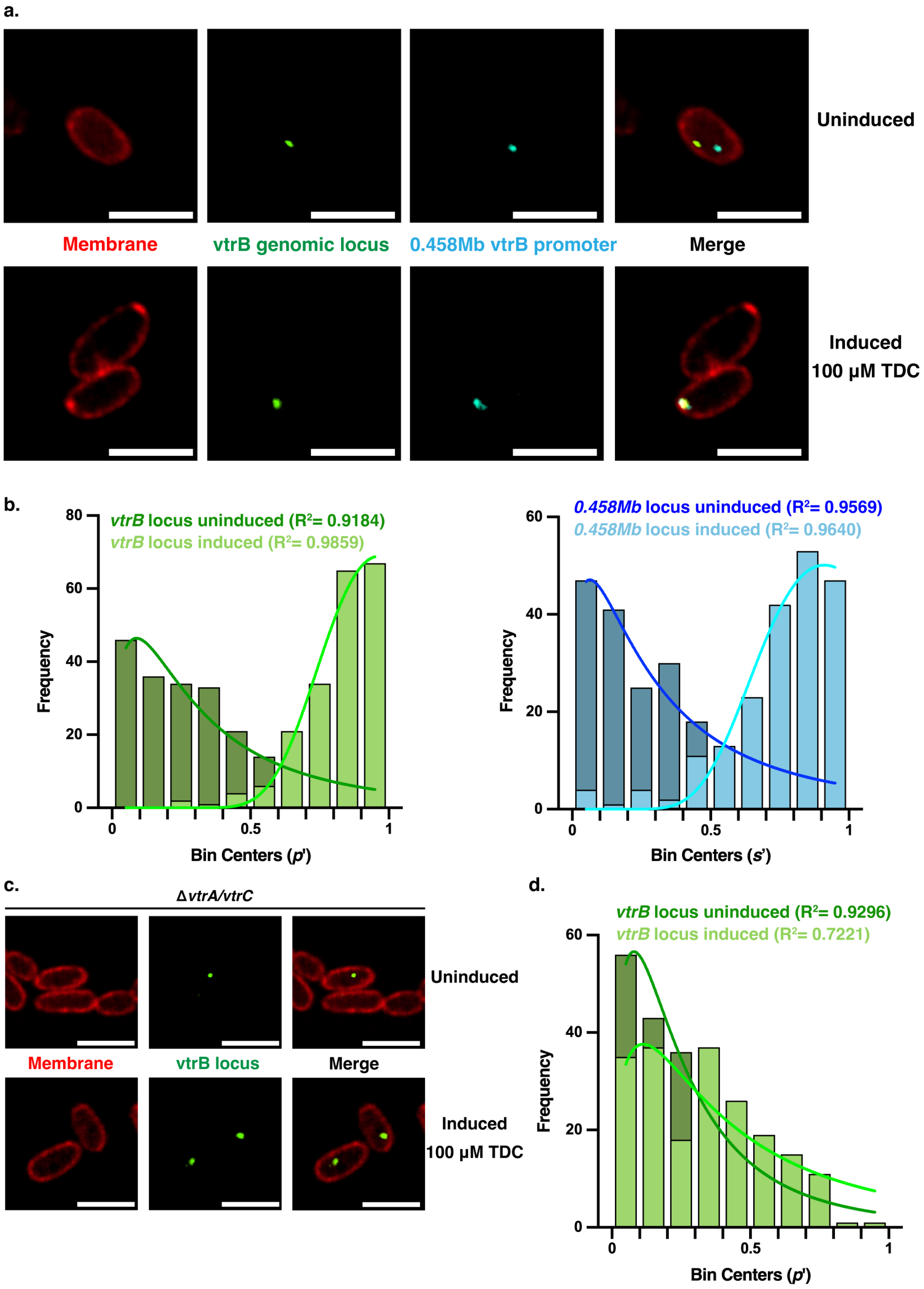


**Supplementary Fig. 2. Bile-activated VtrA/VtrC captures the *vtrB* locus at the membrane via the *vtrB* promoter.**

**a.** Confocal micrographs showing localization of the *vtrB* (green) and neutral + $P_{vpa1348}$ (cyan) loci relative to the membrane (red) in *V. parahaemolyticus* VPKK15 cells cultured in non-

inducing vs. inducing (100 μM TDC) conditions. **b.** Frequency distribution plots with nonlinear regression (lognormal) analyses of normalized *vtrB* loci (p') and neutral + $P_{vpa1348}$ loci (s') distances in *V. parahaemolyticus* VPKK11 cells, cultured as described above. **c.** Confocal micrographs of similarly cultured VPKK18 cells displaying *vtrB* (green) genomic loci proximity to the membrane (red). **d.** Frequency distribution plots with nonlinear regression (lognormal) analyses of normalized *vtrB* loci (p') distances in *V. parahaemolyticus* VPKK18 cells, cultured as described above. **a.** and **c.** Scale bar = 2 μm. **b.** and **d.** N= 200 loci/condition were measured and quantified. $R^2$ = goodness of fit of the regression curves. **a**-**d.** Three independent biological repetitions were performed with similar results. Source data are provided as a Source Data file.

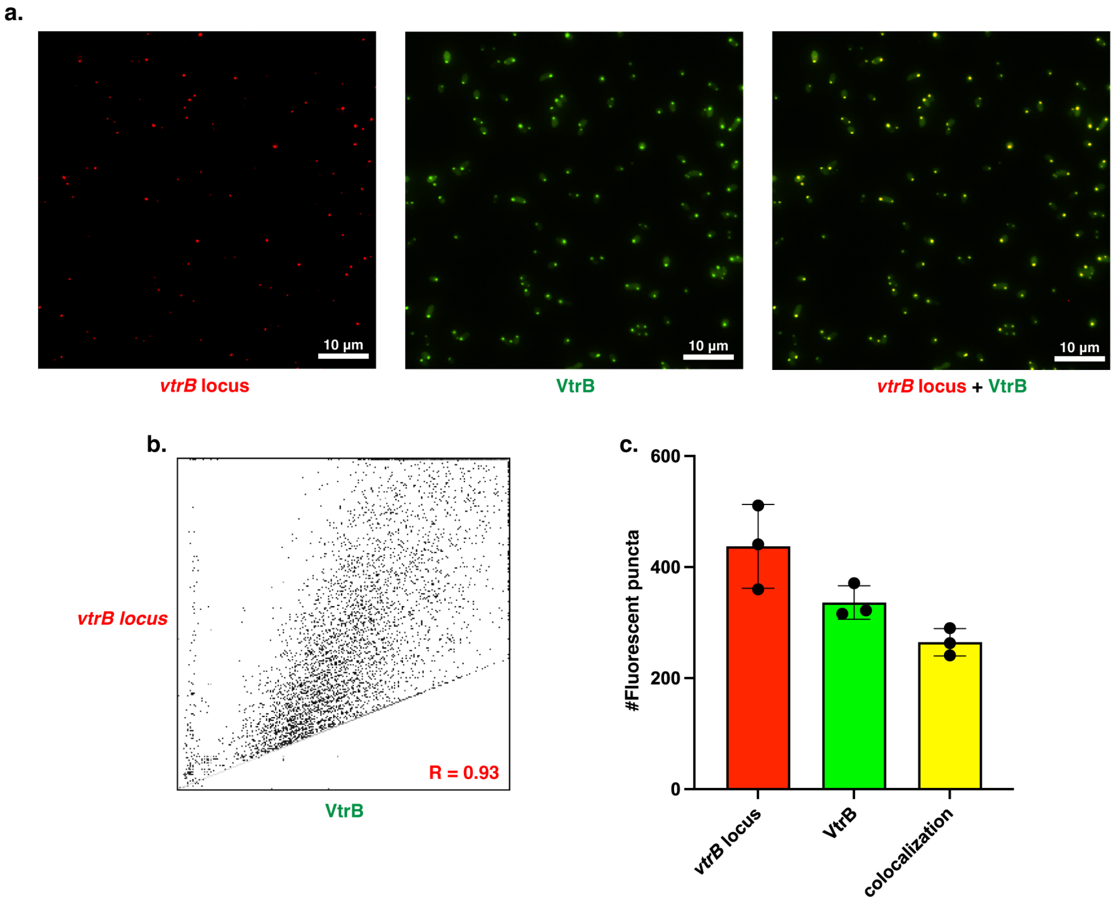


**Supplementary Fig. 3. Bile-induction leads to membrane insertion of VtrB at the site of *vtrB* locus capture.**

**a.** Widefield micrographs of *V. parahaemolyticus* SC295 cells cultured in inducing (100 $\mu$M TDC) conditions, showing global expression and localization of the *vtrB* locus (false-colored red) and VtrB (green). **b.** Scatter plot depicting correlation of VtrB and *vtrB* genomic locus pixel intensities in the widefield micrographs in panel a. R = Pearson's Correlation Coefficient, which is a measure of colocalization. **c.** Histograms representing quantification of total *vtrB* locus (red), total VtrB (green) and *vtrB* locus-VtrB colocalized (yellow) puncta in widefield images of bile-induced SC295 cells. Graph bars represent mean # fluorescent puncta counted +/- SD and black dots represent individual data points for $n$=3 technical replicates (**a** – **c**), where 355 +/- 36 cells were counted, with 2 biological replicates per experiment (**a** – **c**). Source data are provided as a Source Data file.

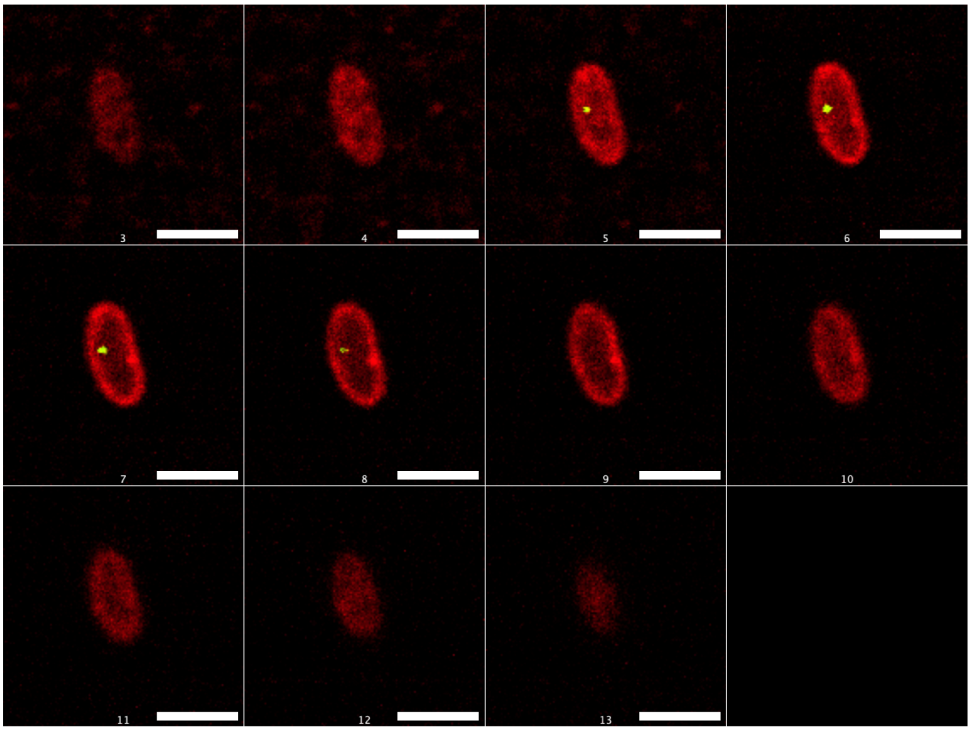


Uninduced

**Supplementary Fig. 4. The *vtrB* locus shows midcell bias in the absence of bile.**

Confocal micrograph z-stacks of *V. parahaemolyticus* VPKK6 (*vpa1343S3C*) cells, corresponding to the upper panel of Fig. 3d, showing localization of the *vtrB* locus (green) relative to the membrane (red). Scale bar = 2 $\mu$m. The images represent z-slices ranging from plane 3 (0.2 $\mu$m) to plane 13 (1.2 $\mu$m) of the z-stack, with a step size of 0.1 $\mu$m.

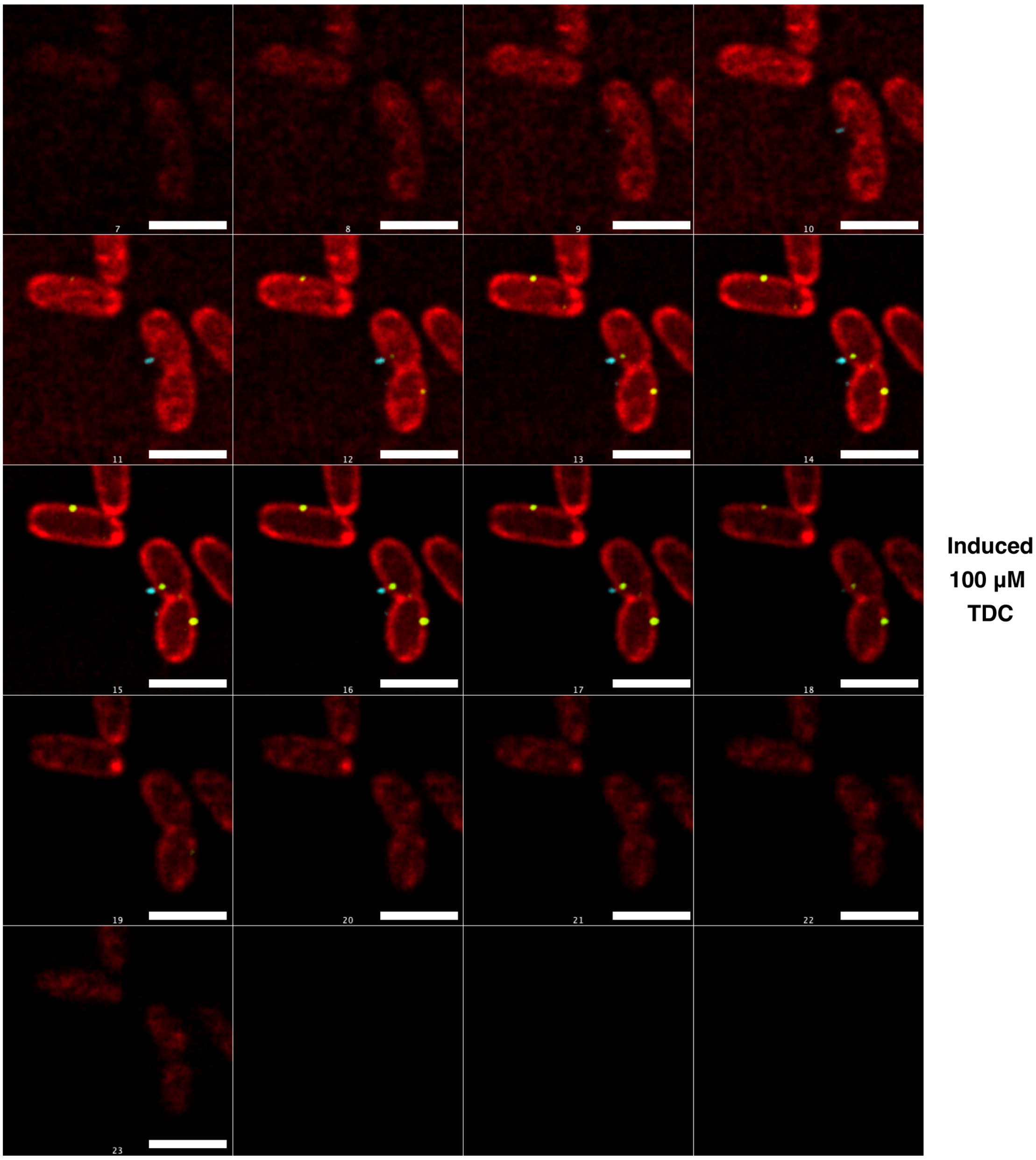


**Supplementary Fig. 5. T3SS2 localizes to the outer membrane adjacent to inner membrane captured *vtrB* locus.**

Confocal micrograph z-stacks of *V. parahaemolyticus* VPKK6 (*vpa1343S3C*) cells cultured in inducing (100 $\mu$M TDC), corresponding to the lower panel of Fig. 3d, showing localization of the T3SS2 needles (false-colored cyan) with the membrane captured *vtrB* locus (green) at the membrane (red). Scale bar = 2 $\mu$m. The images represent z-slices ranging from plane 7 (0.6 $\mu$m) to plane 23 (2.2 $\mu$m) of the z-stack, with a step size of 0.1 $\mu$m.

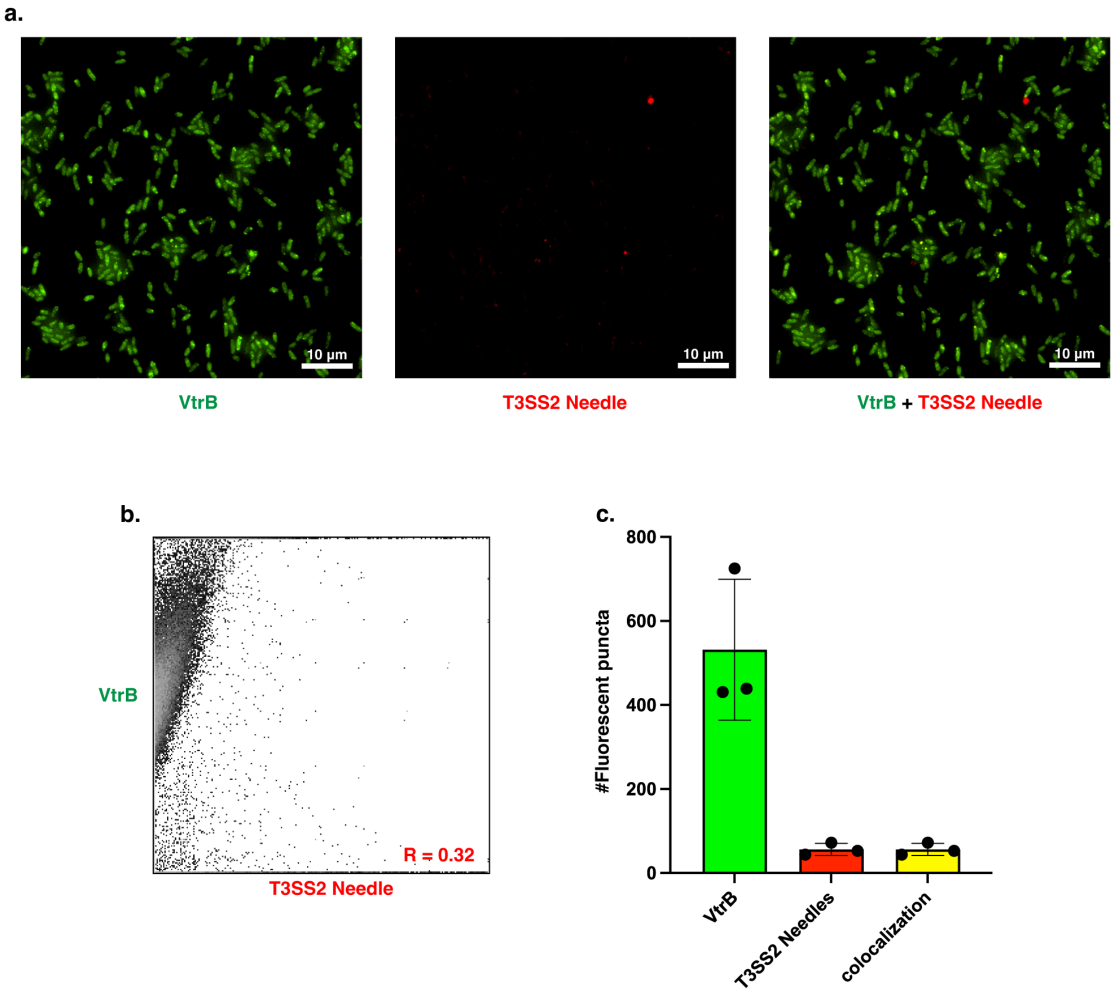


**Supplementary Fig. 6. T3SS2 is assembled on the outer membrane adjacent to inner membrane-inserted VtrB.**

**a.** Widefield micrographs of *V. parahaemolyticus* VPKK19 cells grown in inducing (100 $\mu$M TDC) conditions, depicting localization of VtrB (green) and T3SS2 needles (false-colored red). **b.** Scatter plot showing correlation of VtrB and T3SS2 needle pixel intensities in the widefield micrographs in panel a. R = Pearson's Correlation Coefficient. **c.** Histograms denoting quantification of total VtrB (green), T3SS2 needles (red) and VtrB-T3SS2 colocalized (yellow) puncta in widefield images of TDC-induced cells. Graph bars represent mean # fluorescent puncta counted +/- SD and black dots represent individual data points for $n=3$ technical replicates (**a** – **c**), where 752 +/- 142 cells were counted, with 2 biological replicates per experiment (**a** – **c**). Source data are provided as a Source Data file.

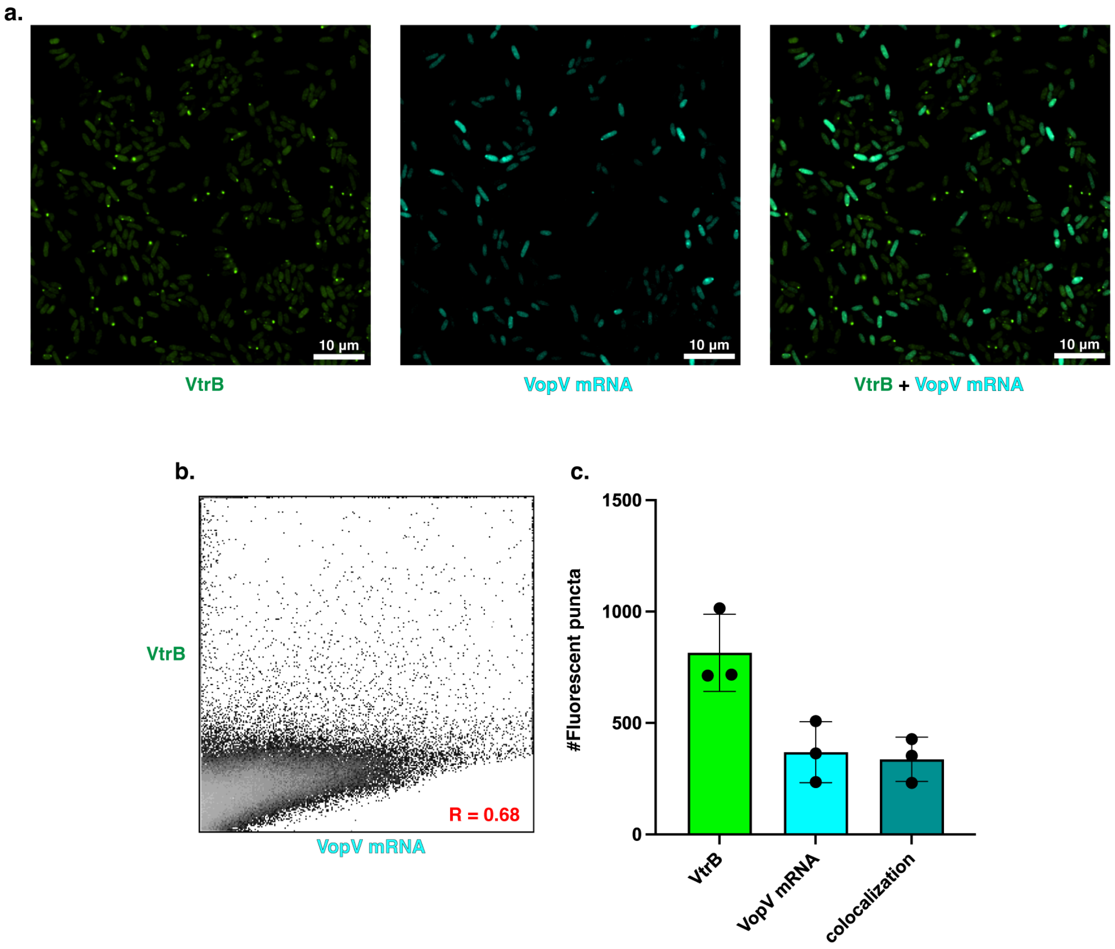


**Supplementary Fig. 7. T3SS2 effectors are transcribed at the site of membrane-inserted VtrB.**

**a.** Widefield micrographs displaying localization of VtrB (green) and VopV mRNA (cyan) in 100 $\mu$M TDC-induced *V. parahaemolyticus* VPKK19 cells **b.** Scatter plot of VtrB and VopV mRNA pixel intensity correlations in the widefield micrographs in panel a. R = Pearson's Correlation Coefficient. **c.** Histograms denoting quantification of total VtrB (green), T3SS2 needles (cyan) and VtrB-T3SS2 colocalized (teal) puncta in widefield images of TDC-induced cells. Graph bars represent mean # fluorescent puncta counted +/- SD and black dots represent individual data points for $n = 3$ technical replicates (**a** – **c**), where 900 +/- 57 cells were counted, with 2 biological replicates per experiment (**a** – **c**). Source data are provided as a Source Data file.

## Supplementary References


1 Zhang, L. *et al.* Type III Effector VopC Mediates Invasion for Vibrio Species. *Cell Reports* **1**, 453-460 (2012). https://doi.org:10.1016/j.celrep.2012.04.004

2 Dalia, A. B. & Dalia, T. N. Spatiotemporal Analysis of DNA Integration during Natural Transformation Reveals a Mode of Nongenetic Inheritance in Bacteria. *Cell* **179**, 1499-1511.e1410 (2019). https://doi.org:10.1016/j.cell.2019.11.021

3 Abudayyeh, O. O. *et al.* RNA targeting with CRISPR-Cas13. *Nature* **550**, 280-284 (2017). https://doi.org:10.1038/nature24049

4 Hocine, S., Raymond, P., Zenklusen, D., Chao, J. A. & Singer, R. H. Single-molecule analysis of gene expression using two-color RNA labeling in live yeast. *Nat Methods* **10**, 119-121 (2013). https://doi.org:10.1038/nmeth.2305

5 Lau, I. F. *et al.* Spatial and temporal organization of replicating Escherichia coli chromosomes. *Mol Microbiol* **49**, 731-743 (2003). https://doi.org:10.1046/j.1365-2958.2003.03640.x
