# Supplementary material for: Membrane-localized expression, production and assembly of Vibrio parahaemolyticus T3SS2 provides evidence for transertion
Source: Nat Commun. 2023 Mar 2;14:1178. doi: 10.1038/s41467-023-36762-z (PMC9977878; doi:10.1038/s41467-023-36762-z)
Supplement: Supplementary file 3 — Reporting Summary [file 41467_2023_36762_MOESM3_ESM.pdf]

nature portfolio

Corresponding author(s): KIM ORTH
Last updated by author(s): Feb 10, 2023

Reporting Summary

Nature Portfolio wishes to improve the reproducibility of the work that we publish. This form provides structure for consistency and transparency in reporting. For further information on Nature Portfolio policies, see our Editorial Policies and the Editorial Policy Checklist.

Statistics

For all statistical analyses, confirm that the following items are present in the figure legend, table legend, main text, or Methods section.

n/a	Confirmed	
☐	☒	The exact sample size (*n*) for each experimental group/condition, given as a discrete number and unit of measurement
☐	☒	A statement on whether measurements were taken from distinct samples or whether the same sample was measured repeatedly
☐	☒	The statistical test(s) used AND whether they are one- or two-sided<br>*Only common tests should be described solely by name; describe more complex techniques in the Methods section.*
☒	☐	A description of all covariates tested
☐	☒	A description of any assumptions or corrections, such as tests of normality and adjustment for multiple comparisons
☐	☒	A full description of the statistical parameters including central tendency (e.g. means) or other basic estimates (e.g. regression coefficient) AND variation (e.g. standard deviation) or associated estimates of uncertainty (e.g. confidence intervals)
☐	☒	For null hypothesis testing, the test statistic (e.g. F, t, r) with confidence intervals, effect sizes, degrees of freedom and P value noted<br>*Give P values as exact values whenever suitable.*
☒	☐	For Bayesian analysis, information on the choice of priors and Markov chain Monte Carlo settings
☒	☐	For hierarchical and complex designs, identification of the appropriate level for tests and full reporting of outcomes
☐	☒	Estimates of effect sizes (e.g. Cohen's *d*, Pearson's *r*), indicating how they were calculated

Our web collection on statistics for biologists contains articles on many of the points above.

Software and code

Policy information about availability of computer code

Data collection	OLYMPUS cellSens Dimension 3.2 (Build 23706) was used for super-resolution and widefield image acquisition and processing. Constrained iterative deconvolution (cellSens TruSight 3D Deconvolution module) optimized for super-resolution microscopy (20 iterations) was performed to clean up image noise. The resultant images were used to calculate linear fluorescence intensity profiles (cellSens Count & Measure module). Constrained iterative deconvolution optimized for widefield microscopy (5 iterations) was performed for noise reduction of widefield images.
Data analysis	Plotting of graphs and statistical analyses of data was performed using Prism 9 version 9.5.0. Pixel intensity correlations and colocalizations coefficient calculations were done using Coloc 2 version 3.0.5. Scatter plot of pixel intensities plotted in ScatterJn version 1.0. Both the Coloc 2 and ScatterJn modules were run in ImageJ2 version 2.9.0 (build 133148d777).

For manuscripts utilizing custom algorithms or software that are central to the research but not yet described in published literature, software must be made available to editors and reviewers. We strongly encourage code deposition in a community repository (e.g. GitHub). See the Nature Portfolio guidelines for submitting code & software for further information.

Data

Policy information about availability of data

All manuscripts must include a data availability statement. This statement should provide the following information, where applicable:

- Accession codes, unique identifiers, or web links for publicly available datasets
- A description of any restrictions on data availability
- For clinical datasets or third party data, please ensure that the statement adheres to our policy

The main data supporting the findings of this study are available within the article and its Supplementary Figures. The source data underlying Figs. 2–4, Supplementary Figs. 1–3 and Supplementary Figs. 6–7 are provided as a Source Data file. Specific data P values are also included within the Source Data file. Additional details on datasets such as raw micrographs and protocols that support the findings of this study will be made available by the corresponding author upon reasonable request. Source data are provided with this paper.

Human research participants

Policy information about studies involving human research participants and Sex and Gender in Research.

Reporting on sex and gender	N/A
Population characteristics	N/A
Recruitment	N/A
Ethics oversight	N/A

Note that full information on the approval of the study protocol must also be provided in the manuscript.

Field-specific reporting

Please select the one below that is the best fit for your research. If you are not sure, read the appropriate sections before making your selection.

☒ Life sciences ☐ Behavioural & social sciences ☐ Ecological, evolutionary & environmental sciences

For a reference copy of the document with all sections, see nature.com/documents/nr-reporting-summary-flat.pdf

Life sciences study design

All studies must disclose on these points even when the disclosure is negative.

Sample size	No sample size calculation was performed. For membrane proximity quantifications, it was determined that 200 distinct puncta for the vtrB genomic loci and/or neutral loci per sample, giving N=200 per replicate, was sufficient to observe statistically significant variations in the population. For the purpose of quantification and analysis of colocalization, the number of cells within the field-of-view of a 100x widefield image (300<N<1000/sample/replicate) were deemed to be sufficient.
Data exclusions	No data was excluded.
Replication	Experiments were performed in triplicates, with at least two biological replicates. All attempts at replication were successful.
Randomization	As membrane proximities of genetic loci are dynamic and fall within a distribution, while determination of colocalization first requires measurement of pixel intensities prior to performing correlation analysis, there was no need for sample randomization.
Blinding	This is molecular microbiology rather than a clinical microbiology study, and therefore blinding was not neccessary.

Reporting for specific materials, systems and methods

We require information from authors about some types of materials, experimental systems and methods used in many studies. Here, indicate whether each material, system or method listed is relevant to your study. If you are not sure if a list item applies to your research, read the appropriate section before selecting a response.

Materials & experimental systems

n/a	Involved in the study	
☐	☒	Antibodies
☐	☒	Eukaryotic cell lines
☒	☐	Palaeontology and archaeology
☒	☐	Animals and other organisms
☒	☐	Clinical data
☒	☐	Dual use research of concern

Methods

n/a	Involved in the study	
☒	☐	ChIP-seq
☒	☐	Flow cytometry
☒	☐	MRI-based neuroimaging

Antibodies

Antibodies used

Primary antibodies: anti-VopL (Rabbit polyclonal, custom order from Strategic Biosolutions, Newark, DE), anti-VtrB (Rat polyclonal, custom order from Thermo Fisher, Project#: 1 WF1568, Material#: HAB2082V), anti-VPA1343 (Rabbit polyclonal, custom order from Thermo Fisher, Project#: 1WF1571, Material#:HAB2016V), anti-GFP[Living Colors A.v. Monoclonal Antibody (JL-8), Takara Bio, Cat#: 632381].
Secondary antibodies: anti-rabbit IgG HRP-conjugated (Donkey polyclonal, Fisher, Cat#: NA934-1ML), anti-Rabbit IgG (H+L) Alexa Fluor 488-5 nm colloidal gold-conjugated (Goat polyclonal, Fisher, Cat#: A-31565), anti-mouse IgG 12 nm colloidal Gold-AffiniPure (Goat polyclonal, Jackson ImmunoResearch Laboratories, Inc., Cat#: 115-205-146, Lot#: 114084).

Validation

Custom antibody for VopL has been validated using western blots and used for prior publications (1 Liverman, A. D. B. et al. Arp2/3-independent assembly of actin by Vibrio type III effector VopL.
Proceedings of the National Academy of Sciences 104, 17117-17122 (2007). https://doi.org:doi:10.1073/pnas.0703196104). Custom antibodies for VPA1343 and VtrB have been validated using western blotting.
Custom antibodies for VtrB and Vpa1343 having titers 12,500 and 200,000, respectively, were verified using ELISA. Serial 1:4 dilutions of serum samples (1:195, 1:781, 1:3,125, 1:12,500, 1:50,000, and 1:200,000) were tested by ELISA in duplicate. Titers are reported as the reciprocal of the dilution factor of the serum samples and are defined as the highest dilution at which the Absorbance (405 nm) remains above the pre-bleed (pre-immune) control value from the same animal. The pre-bleed control value was calculated as the mean absorbance of the pre-bleed dilution series plus 3 standard deviations. Anti-antigen antibodies were detected by indirect ELISA with unconjugated antigens passively coated on plates, probed with anti-IgG-HRP conjugate, and detected with ABTS substrate.
Commercially available antibodies procured from Fisher [anti-rabbit IgG HRP-conjugated and anti-Rabbit IgG (H+L) Alexa Fluor 488-5 nm colloidal gold-conjugated] were validated using a two-part testing approach detailed on their website (https://www.thermofisher.com/us/en/home/life-science/antibodies/invitrogen-antibody-validation.html#2-part-testing-flyer).
Anti-GFP antibodies[Living Colors A.v. Monoclonal Antibody (JL-8)] from Takara Bio was tested by Western blot analysis using lysate made from a HEK 293 cell line stably expressing AcGFP1. After cells were collected and lysed using SDS sample buffer, the lysate (10 µl; equivalent to 35,000 cells) was electrophoresed on a 12% SDS- polyacrylamide gel and transferred to a nitrocellulose membrane. The blot was probed with the Living Colors A.v. Monoclonal Antibody, JL-8 (diluted 1:1,000), followed by a secondary goat anti-mouse antibody conjugated to horseradish peroxidase (HRP). The HRP signal was detected by chemiluminescence. A band of approximately 30 kDa corresponding to AcGFP1 was observed in the lane loaded with the AcGFP1 cell lysate. A band of this size was not detected in the lysate of untransfected HEK 293 cells.
Anti-mouse IgG 12 nm colloidal Gold-AffiniPure antibodies from Jackson ImmunoResearch Laboratories were validated by ELISA detailed on their website (https://www.jacksonimmuno.com/catalog/products/115-205-146).

Eukaryotic cell lines

Policy information about cell lines and Sex and Gender in Research

Cell line source(s)	Hela (ATCC Cat#: CCL-2)
Authentication	The cell line was not authenticated.
Mycoplasma contamination	The cell line was not tested for Mycoplasma contamination.
Commonly misidentified lines (See ICLAC register)	No commonly misidentified cell line was used in the study.
